# 4,4′-Methyl­enebis[*N*-(2-hy­droxy-3-meth­oxy­benzyl­idene)-2,6-diiso­propyl­aniline]

**DOI:** 10.1107/S2414314622007933

**Published:** 2022-08-26

**Authors:** Tulasi Prapakaran, Ramaswamy Murugavel

**Affiliations:** a Department of Chemistry, Indian Institute of Technology Bombay, Powai, Mumbai-400076, India; Howard University, USA

**Keywords:** crystal structure, bis-bidentate bulky Schiff base, hydrogen bonding

## Abstract

In the V-shaped title Schiff base, C_41_H_50_N_2_O_4_, the planes of the benzene rings of the central di­phenyl­methane unit make a dihedral angle of 70.40 (5)°, whereas the planes of the neighbouring benzene and *ortho*-vanilin rings are twisted with respect to one another by dihedral angles of 75.76 (5) and 73.89 (6)°. The Schiff base displays intra­molecular O—H⋯N hydrogen bonds and weak inter­molecular C—H⋯O contacts.

## Abstract 2

In the V-shaped bis-bidentate bulky Schiff base title mol­ecule, the phenolic –OH group forms intra­molecular hydrogen bonds. In the crystal, weak inter­molecular C—H⋯O inter­actions connect the mol­ecules.

## Graphical abstract

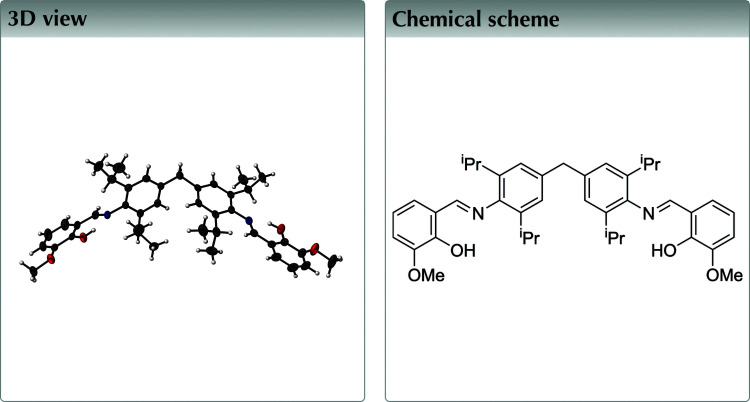

## Structure description

**Figure 1:**
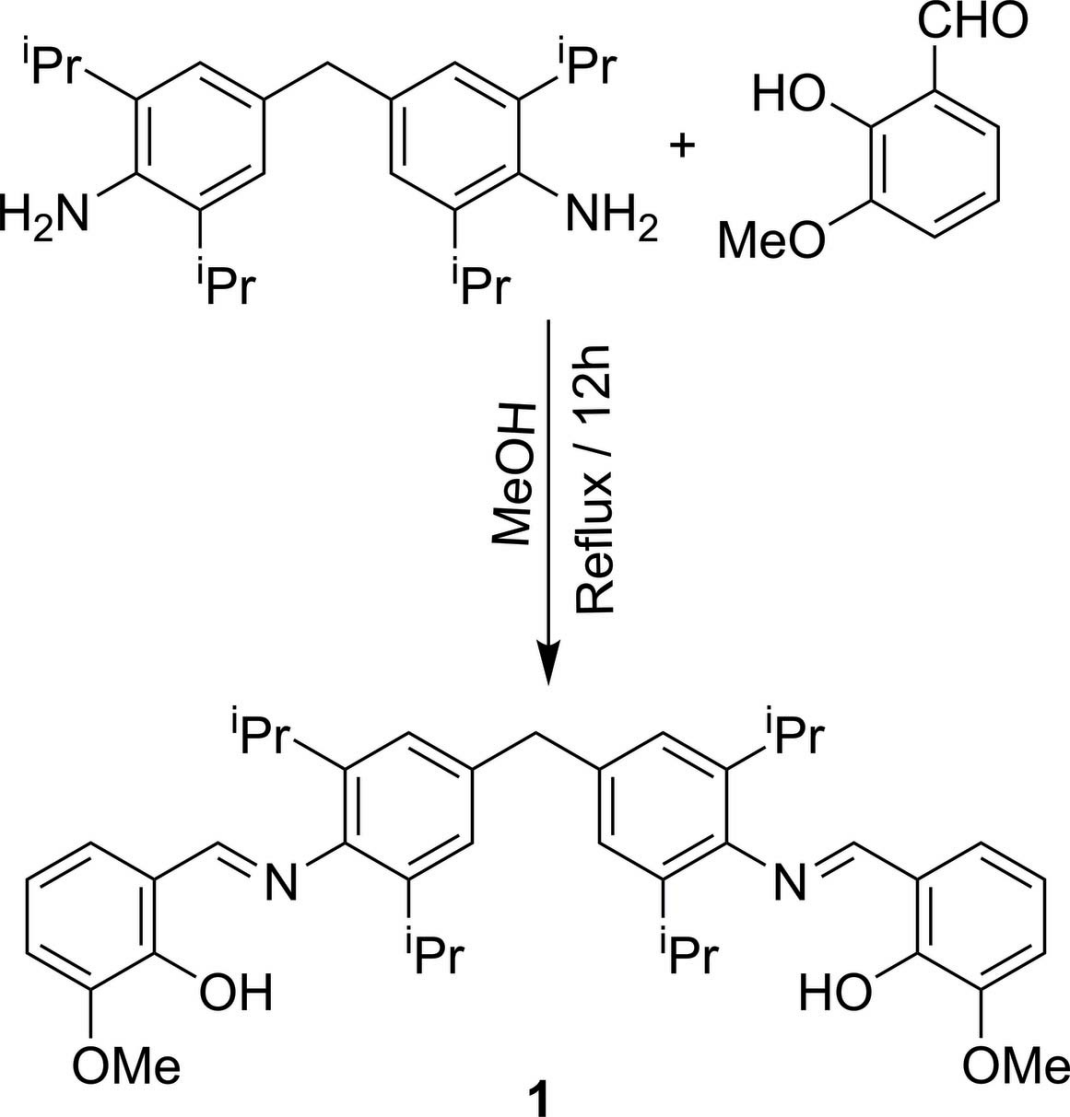
Reaction scheme.

Bis-bidentate Schiff ligands have been widely used as building blocks in metallo-supra­molecular chemistry (Xu *et al.*, 2015 ▸; Chu & Huang, 2007 ▸; Birkedal & Pattison, 2006 ▸). Additionally, these compounds have been employed as thermosetting resins (Lin *et al.*, 2008 ▸). We are inter­ested in such ligands because of their diverse applications in coordination chemistry and their single mol­ecular magnetic and luminescent properties (Cucos *et al.*, 2014 ▸; Habib *et al.*, 2012 ▸; Taneda *et al.*, 2004 ▸, 2009 ▸; Novitchi *et al.*, 2008 ▸). As part of our ongoing studies in this area, we describe here the synthesis and characterization of the title *ortho*-vanillin-based bis-bidentate Schiff base. The reaction of 4,4′–methyl­ene-bis­(2,6–diiso­propyl­anilne) with *o*-vanillin led to the formation of 4,4′-methyl­enebis[*N*-(2-hy­droxy-3-meth­oxy­benzyl­idene)-2,6-diiso­propyl­aniline] (Fig. 1 ▸).

**Figure 2:**
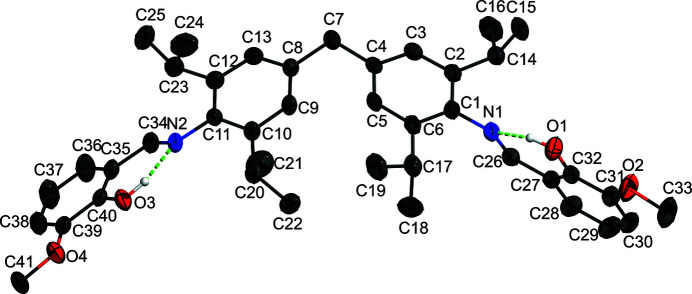
The mol­ecular crystal structure of compound **1**. Ellipsoids represent the 50% probability level. C-bonded H atoms are omitted.

**Figure 3:**
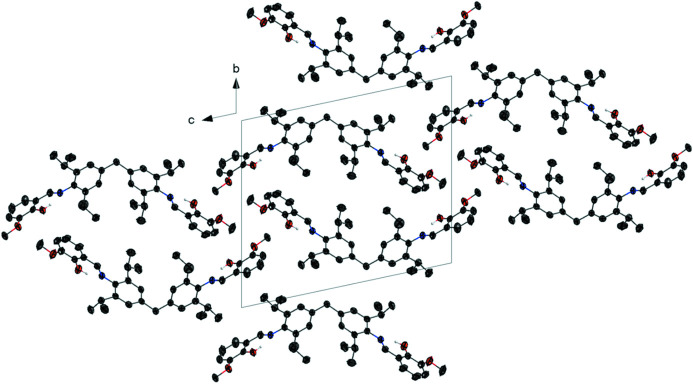
Crystal packing of compound **1** viewed down the *a* axis.

The asymmetric unit of the title mol­ecule contains one mol­ecule (Fig. 2 ▸). The dihedral angle between the planes of the benzene rings bonded to the central methyl­ene group is 70.4 (5)°. The phenyl and *o*-vanillin rings are nearly perpendicular to one another, with dihedral angles of 75.76 (5)° and 73.89 (6)°. The N1—C26 and N2—C34 bond lengths [1.281 (2) and 1.278 (2) Å] support the double-bond nature of the C—N bonds. The mol­ecule exhibits an imine *E* configuration with C1—N1—C26—C27 and C11—N2—C34—C35 torsion angles of 179.37 (16) and 177.85 (15)°, respectively. In the mol­ecule, atoms N1 and N2 of the imine moieties serve as hydrogen-bond acceptors, with adjacent phenol groups forming an intra­molecular O—H⋯N hydrogen bond. The closest inter­molecular contact is C41—H41*B*⋯O2 (at *x*, *y*, 1 + *z*), resulting in zigzag chain formation (Table 1 ▸). Fig. 3 ▸ depicts the packing of the title compound.

## Synthesis and crystallization

4,4′–Methyl­ene-bis­(2,6–diiso­propyl­anilne) (1 g, 2.72 mmol) and *o*-vanilin (0.870 g, 5.72 mmol) were dissolved in methanol (40 ml) and heated under reflux overnight, resulting in a yellow solution that was filtered and crystallized by slow evaporation at room temperature. The crystals were filtered and washed with cold methanol and dried under reduced pressure (985 mg, 56% yield, m.p. 143°C). Analysis calculated for (%) C_41_H_50_N_2_O_4_: C, 77.57; H, 7.94; N, 4.41. Found: C, 76.99; H, 7.96; N, 4.18. FT–IR (KBr pellet) 3447 cm^−1^ [*m*, ν (O—H)], 2959 cm^−1^ [*s*, ν (Ar—H)], 1621 cm^−1^ [*s*, ν (C=N)]. ^1^H NMR (CDCl_3_, 500 MHz) δ p.p.m. 1.15 (*d*, 12H), 3.0 (*m*, 2H, CHMe_2_), 4.0 (*s*, 3H, OMe), 4.01 (*s*, 2H, CH_2_), 6.89–6.97 (*m*, 3H), 7.03 (*s*, 2H), 8.30 (*s*, 1H, CH=N), 13.62 (*br*, 1H, OH). ^13^C NMR (CDCl_3_, 125 MHz) δ p.p.m. 23.7, 28.3, 41.8, 56.3, 114.7, 118.7, 123.7, 138.2, 144, 148.7, 151.6, 167.

## Refinement

Crystal data, data collection and structure refinement details are summarized in Table 2 ▸.

## Supplementary Material

Crystal structure: contains datablock(s) I. DOI: 10.1107/S2414314622007933/bv4044sup1.cif

Supporting information file. DOI: 10.1107/S2414314622007933/bv4044Isup2.cml

CCDC reference: 2195233

Additional supporting information:  crystallographic information; 3D view; checkCIF report

## Figures and Tables

**Table 1:** Hydrogen-bond geometry (Å, °)

*D*—H⋯*A*	*D*—H	H⋯*A*	*D*⋯*A*	*D*—H⋯*A*
O3—H3⋯N2	0.87 (2)	1.79 (2)	2.5912 (17)	153 (2)
O1—H1⋯N1	0.87 (2)	1.80 (2)	2.5991 (19)	153 (2)
C41—H41*B*⋯O2^i^	0.98	2.49	3.452 (3)	168

Symmetry code: (i) 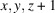 .

**Table 2:** Experimental details

Crystal data	
Chemical formula	C_41_H_50_N_2_O_4_
*M*_r_	634.83
Crystal system, space group	Triclinic, *P* 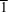
Temperature (K)	150
*a*, *b*, *c* (Å)	6.0155 (1), 16.5935 (3), 19.2093 (3)
α, β, γ (°)	101.541 (2), 97.341 (2), 92.928 (2)
*V* (Å^3^)	1857.47 (6)
*Z*	2
Radiation type	Mo *K*α
μ (mm^−1^)	0.07
Crystal size (mm)	0.45 × 0.14 × 0.10

Data collection	
Diffractometer	Rigaku Saturn 724+
Absorption correction	Multi-scan (*CrysAlis PRO*; Rigaku OD, 2017 ▸)
*T*_min_, *T*_max_	0.701, 1.000
No. of measured, independent and observed [*I* > 2σ(*I*)] reflections	14358, 6528, 5207
*R*_int_	0.020
(sin θ/λ)_max_ (Å^−1^)	0.595

Refinement	
*R*[*F* ^2^ > 2σ(*F* ^2^)], *wR*(*F* ^2^), *S*	0.048, 0.126, 1.03
No. of reflections	6528
No. of parameters	440
No. of restraints	2
H-atom treatment	H atoms treated by a mixture of independent and constrained refinement
Δρ_max_, Δρ_min_ (e Å^−3^)	0.36, −0.17

Computer programs: *CrysAlis PRO* (Rigaku OD, 2017 ▸), *SHELXT2015* (Sheldrick, 2015*a* ▸), *SHELXL2015* (Sheldrick, 2015*b* ▸), *OLEX2* (Dolomanov *et al.*, 2009 ▸) and *publCIF* (Westrip, 2010 ▸).

## full crystallographic data

### Crystal data

**Table:**

C_41_H_50_N_2_O_4_	*Z* = 2
*M**_r_* = 634.83	*F*(000) = 684
Triclinic, *P*1	*D*_x_ = 1.135 Mg m^−^^3^
*a* = 6.0155 (1) Å	Mo *K*α radiation, λ = 0.71073 Å
*b* = 16.5935 (3) Å	Cell parameters from 9193 reflections
*c* = 19.2093 (3) Å	θ = 2.3–31.1°
α = 101.541 (2)°	µ = 0.07 mm^−^^1^
β = 97.341 (2)°	*T* = 150 K
γ = 92.928 (2)°	Needle, yellow
*V* = 1857.47 (6) Å^3^	0.45 × 0.14 × 0.1 mm

### Data collection

**Table:**

Rigaku Saturn 724+ diffractometer	6528 independent reflections
Radiation source: fine-focus sealed X-ray tube, Enhance (Mo) X-ray Source	5207 reflections with *I* > 2σ(*I*)
Graphite monochromator	*R*_int_ = 0.020
ω scans	θ_max_ = 25.0°, θ_min_ = 2.5°
Absorption correction: multi-scan (CrysAlisPro; Rigaku OD, 2017)	*h* = −7→7
*T*_min_ = 0.701, *T*_max_ = 1.000	*k* = −19→15
14358 measured reflections	*l* = −21→22

### Refinement

**Table:**

Refinement on *F*^2^	Primary atom site location: dual
Least-squares matrix: full	Hydrogen site location: mixed
*R*[*F*^2^ > 2σ(*F*^2^)] = 0.048	H atoms treated by a mixture of independent and constrained refinement
*wR*(*F*^2^) = 0.126	*w* = 1/[σ^2^(*F*_o_^2^) + (0.0537*P*)^2^ + 0.623*P*] where *P* = (*F*_o_^2^ + 2*F*_c_^2^)/3
*S* = 1.03	(Δ/σ)_max_ = 0.001
6528 reflections	Δρ_max_ = 0.36 e Å^−^^3^
440 parameters	Δρ_min_ = −0.17 e Å^−^^3^
2 restraints	

### Special details

**Table:**

Geometry. All esds (except the esd in the dihedral angle between two l.s. planes) are estimated using the full covariance matrix. The cell esds are taken into account individually in the estimation of esds in distances, angles and torsion angles; correlations between esds in cell parameters are only used when they are defined by crystal symmetry. An approximate (isotropic) treatment of cell esds is used for estimating esds involving l.s. planes.

### Fractional atomic coordinates and isotropic or equivalent isotropic displacement parameters (Å^2^)

**Table:**

	*x*	*y*	*z*	*U*_iso_*/*U*_eq_	
O3	1.0025 (2)	0.74747 (8)	0.94420 (6)	0.0440 (3)	
H3	0.951 (3)	0.7684 (12)	0.9080 (9)	0.053*	
O1	0.7058 (2)	0.60969 (8)	0.22598 (7)	0.0516 (4)	
H1	0.681 (4)	0.6413 (12)	0.2656 (9)	0.062*	
N2	0.7464 (2)	0.82044 (8)	0.86122 (7)	0.0331 (3)	
O4	1.0694 (3)	0.68864 (8)	1.06103 (7)	0.0578 (4)	
N1	0.5193 (3)	0.67089 (8)	0.33770 (7)	0.0382 (3)	
O2	0.6886 (3)	0.49933 (10)	0.10588 (8)	0.0728 (5)	
C11	0.6910 (3)	0.84537 (10)	0.79454 (8)	0.0319 (4)	
C1	0.5145 (3)	0.73131 (10)	0.40200 (8)	0.0359 (4)	
C40	0.8509 (3)	0.76246 (9)	0.99044 (8)	0.0354 (4)	
C12	0.8185 (3)	0.91305 (10)	0.78247 (9)	0.0365 (4)	
C10	0.5268 (3)	0.79864 (10)	0.74104 (8)	0.0355 (4)	
C34	0.6145 (3)	0.83185 (10)	0.90880 (8)	0.0371 (4)	
H34	0.481609	0.858880	0.899791	0.044*	
C6	0.6688 (3)	0.72676 (10)	0.46248 (8)	0.0373 (4)	
C35	0.6612 (3)	0.80476 (10)	0.97640 (8)	0.0368 (4)	
C39	0.8850 (3)	0.73198 (10)	1.05412 (9)	0.0419 (4)	
C2	0.3717 (3)	0.79565 (10)	0.40243 (9)	0.0389 (4)	
C26	0.3601 (3)	0.61353 (10)	0.31618 (9)	0.0406 (4)	
H26	0.240549	0.611575	0.343812	0.049*	
C20	0.4027 (3)	0.72003 (11)	0.74959 (9)	0.0415 (4)	
H20	0.465958	0.708075	0.796857	0.050*	
C27	0.3567 (3)	0.55148 (10)	0.25099 (9)	0.0437 (5)	
C17	0.8142 (3)	0.65446 (11)	0.46037 (9)	0.0429 (4)	
H17	0.850517	0.638445	0.410326	0.052*	
C13	0.7696 (3)	0.93566 (10)	0.71661 (9)	0.0443 (5)	
H13	0.854035	0.981442	0.707478	0.053*	
C9	0.4843 (3)	0.82531 (11)	0.67685 (9)	0.0440 (4)	
H9	0.370611	0.795636	0.640563	0.053*	
C32	0.5288 (3)	0.55181 (10)	0.20858 (9)	0.0450 (5)	
C5	0.6753 (3)	0.78841 (11)	0.52375 (9)	0.0445 (5)	
H5	0.778531	0.786489	0.565046	0.053*	
C8	0.6012 (4)	0.89338 (10)	0.66378 (9)	0.0479 (5)	
C14	0.2141 (3)	0.80248 (11)	0.33507 (9)	0.0455 (5)	
H14	0.156626	0.745187	0.309572	0.055*	
C23	1.0007 (3)	0.96050 (11)	0.84052 (10)	0.0430 (4)	
H23	1.060884	0.920571	0.869481	0.052*	
C4	0.5351 (4)	0.85284 (10)	0.52639 (9)	0.0484 (5)	
C38	0.7348 (4)	0.74707 (12)	1.10290 (10)	0.0541 (5)	
H38	0.757654	0.726711	1.145902	0.065*	
C3	0.3853 (4)	0.85535 (11)	0.46560 (9)	0.0461 (5)	
H3A	0.289154	0.899146	0.467117	0.055*	
C22	0.4396 (3)	0.64662 (11)	0.69073 (10)	0.0492 (5)	
H22A	0.375698	0.656181	0.643808	0.074*	
H22B	0.365593	0.596154	0.699171	0.074*	
H22C	0.601106	0.640609	0.691609	0.074*	
C25	0.9011 (4)	1.02789 (12)	0.89148 (11)	0.0559 (5)	
H25A	0.837631	1.067768	0.864671	0.084*	
H25B	1.019478	1.056175	0.929668	0.084*	
H25C	0.782346	1.002839	0.912677	0.084*	
C15	0.3467 (4)	0.83974 (13)	0.28492 (9)	0.0506 (5)	
H15A	0.414913	0.894536	0.309715	0.076*	
H15B	0.245340	0.844596	0.242036	0.076*	
H15C	0.465146	0.804004	0.270828	0.076*	
C36	0.5113 (4)	0.81944 (14)	1.02708 (10)	0.0561 (5)	
H36	0.382589	0.848622	1.017936	0.067*	
C19	1.0370 (3)	0.67380 (14)	0.51052 (11)	0.0572 (5)	
H19A	1.115965	0.723219	0.501878	0.086*	
H19B	1.130050	0.627070	0.501414	0.086*	
H19C	1.008032	0.683539	0.560462	0.086*	
C18	0.6850 (4)	0.57976 (12)	0.47611 (12)	0.0582 (5)	
H18A	0.649514	0.592890	0.525320	0.087*	
H18B	0.777577	0.532586	0.470693	0.087*	
H18C	0.545238	0.565974	0.442407	0.087*	
C31	0.5186 (4)	0.49171 (12)	0.14498 (10)	0.0564 (6)	
C30	0.3394 (5)	0.43240 (13)	0.12604 (12)	0.0692 (7)	
H30	0.333160	0.391143	0.083543	0.083*	
C16	0.0106 (4)	0.85078 (14)	0.35006 (12)	0.0578 (5)	
H16A	−0.068439	0.827835	0.384144	0.087*	
H16B	−0.091097	0.846769	0.305168	0.087*	
H16C	0.060441	0.908807	0.370516	0.087*	
C24	1.1994 (4)	0.99674 (13)	0.81108 (13)	0.0617 (6)	
H24A	1.256611	0.953185	0.777110	0.093*	
H24B	1.319035	1.019800	0.850773	0.093*	
H24C	1.149822	1.040428	0.786503	0.093*	
C41	1.1111 (5)	0.65391 (13)	1.12360 (10)	0.0706 (7)	
H41A	1.248975	0.624954	1.122168	0.106*	
H41B	0.984325	0.614930	1.124753	0.106*	
H41C	1.128626	0.698107	1.166670	0.106*	
C37	0.5509 (4)	0.79164 (15)	1.08980 (11)	0.0638 (6)	
H37	0.451395	0.803020	1.124549	0.077*	
C28	0.1750 (4)	0.49100 (12)	0.22983 (12)	0.0594 (6)	
H28	0.056929	0.490900	0.258265	0.071*	
C29	0.1685 (5)	0.43223 (13)	0.16819 (14)	0.0733 (7)	
H29	0.046251	0.391143	0.154217	0.088*	
C7	0.5468 (5)	0.92094 (12)	0.59319 (10)	0.0707 (7)	
H7A	0.662509	0.964598	0.590916	0.085*	
H7B	0.400484	0.945705	0.592621	0.085*	
C21	0.1526 (4)	0.73049 (15)	0.75052 (13)	0.0664 (6)	
H21A	0.132640	0.773893	0.791564	0.100*	
H21B	0.076116	0.678439	0.754658	0.100*	
H21C	0.088405	0.745873	0.705952	0.100*	
C33	0.6978 (6)	0.43475 (17)	0.04340 (12)	0.0931 (10)	
H33A	0.562963	0.433193	0.008493	0.140*	
H33B	0.705075	0.381452	0.057866	0.140*	
H33C	0.831693	0.445924	0.021586	0.140*	

### Atomic displacement parameters (Å^2^)

**Table:**

	*U*^11^	*U*^22^	*U*^33^	*U*^12^	*U*^13^	*U*^23^
O3	0.0572 (8)	0.0496 (7)	0.0265 (6)	0.0123 (6)	0.0014 (6)	0.0118 (5)
O1	0.0671 (9)	0.0487 (8)	0.0317 (7)	−0.0023 (7)	0.0012 (6)	−0.0034 (6)
N2	0.0405 (8)	0.0335 (7)	0.0220 (7)	0.0002 (6)	−0.0033 (6)	0.0037 (5)
O4	0.0913 (11)	0.0533 (8)	0.0309 (7)	0.0170 (8)	−0.0017 (7)	0.0175 (6)
N1	0.0505 (9)	0.0356 (8)	0.0252 (7)	−0.0078 (7)	−0.0046 (6)	0.0073 (6)
O2	0.1026 (13)	0.0644 (10)	0.0392 (8)	0.0231 (9)	−0.0028 (9)	−0.0140 (7)
C11	0.0403 (10)	0.0314 (8)	0.0231 (8)	0.0068 (7)	0.0005 (7)	0.0048 (6)
C1	0.0474 (10)	0.0339 (9)	0.0238 (8)	−0.0136 (8)	−0.0004 (7)	0.0074 (7)
C40	0.0528 (11)	0.0272 (8)	0.0206 (8)	−0.0070 (7)	−0.0016 (7)	−0.0014 (6)
C12	0.0456 (10)	0.0312 (8)	0.0308 (9)	0.0059 (7)	0.0013 (8)	0.0039 (7)
C10	0.0440 (10)	0.0347 (9)	0.0253 (8)	0.0049 (7)	−0.0012 (7)	0.0035 (7)
C34	0.0405 (10)	0.0394 (9)	0.0271 (8)	0.0012 (8)	−0.0028 (8)	0.0021 (7)
C6	0.0467 (10)	0.0373 (9)	0.0269 (8)	−0.0122 (8)	−0.0015 (7)	0.0123 (7)
C35	0.0463 (10)	0.0367 (9)	0.0227 (8)	−0.0068 (8)	−0.0007 (7)	0.0010 (7)
C39	0.0659 (13)	0.0292 (9)	0.0247 (8)	−0.0065 (8)	−0.0062 (8)	0.0023 (7)
C2	0.0539 (11)	0.0337 (9)	0.0279 (9)	−0.0103 (8)	−0.0018 (8)	0.0118 (7)
C26	0.0509 (11)	0.0346 (9)	0.0340 (9)	−0.0047 (8)	−0.0066 (8)	0.0120 (7)
C20	0.0504 (11)	0.0399 (10)	0.0304 (9)	−0.0043 (8)	−0.0043 (8)	0.0068 (7)
C27	0.0602 (12)	0.0292 (9)	0.0358 (9)	−0.0024 (8)	−0.0153 (9)	0.0084 (7)
C17	0.0464 (11)	0.0505 (11)	0.0301 (9)	−0.0055 (8)	−0.0015 (8)	0.0109 (8)
C13	0.0710 (13)	0.0282 (9)	0.0331 (9)	0.0016 (8)	0.0050 (9)	0.0069 (7)
C9	0.0633 (12)	0.0355 (9)	0.0264 (9)	0.0033 (8)	−0.0115 (8)	0.0015 (7)
C32	0.0642 (13)	0.0312 (9)	0.0330 (9)	0.0043 (9)	−0.0162 (9)	0.0055 (7)
C5	0.0675 (13)	0.0390 (10)	0.0237 (8)	−0.0121 (9)	−0.0084 (8)	0.0124 (7)
C8	0.0873 (15)	0.0297 (9)	0.0239 (9)	0.0063 (9)	−0.0018 (9)	0.0045 (7)
C14	0.0637 (13)	0.0357 (9)	0.0329 (9)	−0.0065 (9)	−0.0103 (9)	0.0108 (7)
C23	0.0467 (11)	0.0372 (9)	0.0414 (10)	−0.0026 (8)	−0.0061 (8)	0.0086 (8)
C4	0.0871 (15)	0.0305 (9)	0.0255 (9)	−0.0078 (9)	−0.0034 (9)	0.0113 (7)
C38	0.0837 (16)	0.0504 (11)	0.0261 (9)	−0.0100 (11)	0.0034 (10)	0.0096 (8)
C3	0.0752 (14)	0.0320 (9)	0.0307 (9)	−0.0014 (9)	−0.0002 (9)	0.0118 (7)
C22	0.0581 (12)	0.0363 (10)	0.0476 (11)	−0.0005 (9)	−0.0047 (9)	0.0046 (8)
C25	0.0630 (14)	0.0471 (11)	0.0463 (11)	−0.0044 (10)	−0.0065 (10)	−0.0063 (9)
C15	0.0654 (13)	0.0596 (12)	0.0289 (9)	0.0106 (10)	0.0017 (9)	0.0161 (8)
C36	0.0599 (13)	0.0714 (14)	0.0363 (10)	0.0052 (11)	0.0093 (10)	0.0082 (10)
C19	0.0514 (12)	0.0735 (14)	0.0452 (11)	−0.0060 (10)	−0.0057 (9)	0.0193 (10)
C18	0.0619 (14)	0.0443 (11)	0.0690 (14)	−0.0009 (10)	−0.0026 (11)	0.0222 (10)
C31	0.0854 (16)	0.0401 (11)	0.0355 (10)	0.0170 (11)	−0.0143 (11)	0.0001 (8)
C30	0.108 (2)	0.0358 (11)	0.0472 (12)	0.0088 (12)	−0.0302 (14)	−0.0061 (9)
C16	0.0529 (13)	0.0660 (13)	0.0571 (13)	−0.0065 (10)	0.0005 (10)	0.0264 (11)
C24	0.0530 (13)	0.0530 (12)	0.0765 (15)	−0.0077 (10)	0.0031 (11)	0.0139 (11)
C41	0.129 (2)	0.0532 (12)	0.0291 (10)	0.0189 (13)	−0.0073 (12)	0.0165 (9)
C37	0.0763 (16)	0.0821 (16)	0.0348 (11)	−0.0007 (13)	0.0162 (11)	0.0128 (10)
C28	0.0726 (15)	0.0379 (10)	0.0572 (13)	−0.0126 (10)	−0.0186 (11)	0.0069 (9)
C29	0.097 (2)	0.0386 (12)	0.0668 (15)	−0.0155 (12)	−0.0330 (15)	0.0029 (11)
C7	0.144 (2)	0.0361 (10)	0.0273 (10)	0.0050 (12)	−0.0084 (12)	0.0081 (8)
C21	0.0582 (14)	0.0677 (14)	0.0706 (15)	−0.0051 (11)	0.0169 (12)	0.0056 (12)
C33	0.141 (3)	0.0810 (18)	0.0415 (13)	0.0436 (18)	−0.0069 (15)	−0.0201 (12)

### Geometric parameters (Å, º)

**Table:**

O3—H3	0.867 (15)	C14—C16	1.524 (3)
O3—C40	1.351 (2)	C23—H23	1.0000
O1—H1	0.867 (15)	C23—C25	1.532 (3)
O1—C32	1.358 (2)	C23—C24	1.532 (3)
N2—C11	1.4283 (19)	C4—C3	1.390 (3)
N2—C34	1.278 (2)	C4—C7	1.522 (2)
O4—C39	1.359 (2)	C38—H38	0.9500
O4—C41	1.433 (2)	C38—C37	1.386 (3)
N1—C1	1.431 (2)	C3—H3A	0.9500
N1—C26	1.281 (2)	C22—H22A	0.9800
O2—C31	1.359 (3)	C22—H22B	0.9800
O2—C33	1.449 (2)	C22—H22C	0.9800
C11—C12	1.402 (2)	C25—H25A	0.9800
C11—C10	1.406 (2)	C25—H25B	0.9800
C1—C6	1.408 (2)	C25—H25C	0.9800
C1—C2	1.404 (3)	C15—H15A	0.9800
C40—C35	1.395 (3)	C15—H15B	0.9800
C40—C39	1.410 (2)	C15—H15C	0.9800
C12—C13	1.391 (2)	C36—H36	0.9500
C12—C23	1.521 (2)	C36—C37	1.372 (3)
C10—C20	1.518 (2)	C19—H19A	0.9800
C10—C9	1.390 (2)	C19—H19B	0.9800
C34—H34	0.9500	C19—H19C	0.9800
C34—C35	1.456 (2)	C18—H18A	0.9800
C6—C17	1.517 (3)	C18—H18B	0.9800
C6—C5	1.391 (2)	C18—H18C	0.9800
C35—C36	1.405 (3)	C31—C30	1.382 (3)
C39—C38	1.379 (3)	C30—H30	0.9500
C2—C14	1.530 (2)	C30—C29	1.388 (4)
C2—C3	1.395 (2)	C16—H16A	0.9800
C26—H26	0.9500	C16—H16B	0.9800
C26—C27	1.450 (2)	C16—H16C	0.9800
C20—H20	1.0000	C24—H24A	0.9800
C20—C22	1.534 (2)	C24—H24B	0.9800
C20—C21	1.525 (3)	C24—H24C	0.9800
C27—C32	1.397 (3)	C41—H41A	0.9800
C27—C28	1.406 (3)	C41—H41B	0.9800
C17—H17	1.0000	C41—H41C	0.9800
C17—C19	1.527 (3)	C37—H37	0.9500
C17—C18	1.531 (3)	C28—H28	0.9500
C13—H13	0.9500	C28—C29	1.370 (3)
C13—C8	1.388 (3)	C29—H29	0.9500
C9—H9	0.9500	C7—H7A	0.9900
C9—C8	1.382 (3)	C7—H7B	0.9900
C32—C31	1.406 (3)	C21—H21A	0.9800
C5—H5	0.9500	C21—H21B	0.9800
C5—C4	1.392 (3)	C21—H21C	0.9800
C8—C7	1.518 (2)	C33—H33A	0.9800
C14—H14	1.0000	C33—H33B	0.9800
C14—C15	1.527 (3)	C33—H33C	0.9800
			
C40—O3—H3	105.0 (13)	C2—C3—H3A	118.9
C32—O1—H1	104.8 (15)	C4—C3—C2	122.13 (18)
C34—N2—C11	120.48 (14)	C4—C3—H3A	118.9
C39—O4—C41	117.77 (17)	C20—C22—H22A	109.5
C26—N1—C1	120.19 (15)	C20—C22—H22B	109.5
C31—O2—C33	117.5 (2)	C20—C22—H22C	109.5
C12—C11—N2	117.70 (14)	H22A—C22—H22B	109.5
C12—C11—C10	121.75 (14)	H22A—C22—H22C	109.5
C10—C11—N2	120.32 (14)	H22B—C22—H22C	109.5
C6—C1—N1	117.43 (16)	C23—C25—H25A	109.5
C2—C1—N1	120.55 (15)	C23—C25—H25B	109.5
C2—C1—C6	121.87 (15)	C23—C25—H25C	109.5
O3—C40—C35	122.38 (14)	H25A—C25—H25B	109.5
O3—C40—C39	118.06 (16)	H25A—C25—H25C	109.5
C35—C40—C39	119.54 (16)	H25B—C25—H25C	109.5
C11—C12—C23	120.51 (15)	C14—C15—H15A	109.5
C13—C12—C11	117.75 (16)	C14—C15—H15B	109.5
C13—C12—C23	121.73 (16)	C14—C15—H15C	109.5
C11—C10—C20	123.08 (14)	H15A—C15—H15B	109.5
C9—C10—C11	117.40 (16)	H15A—C15—H15C	109.5
C9—C10—C20	119.48 (15)	H15B—C15—H15C	109.5
N2—C34—H34	118.9	C35—C36—H36	120.0
N2—C34—C35	122.13 (16)	C37—C36—C35	120.0 (2)
C35—C34—H34	118.9	C37—C36—H36	120.0
C1—C6—C17	120.00 (15)	C17—C19—H19A	109.5
C5—C6—C1	117.76 (16)	C17—C19—H19B	109.5
C5—C6—C17	122.20 (15)	C17—C19—H19C	109.5
C40—C35—C34	120.49 (15)	H19A—C19—H19B	109.5
C40—C35—C36	119.71 (16)	H19A—C19—H19C	109.5
C36—C35—C34	119.78 (17)	H19B—C19—H19C	109.5
O4—C39—C40	114.75 (16)	C17—C18—H18A	109.5
O4—C39—C38	125.72 (16)	C17—C18—H18B	109.5
C38—C39—C40	119.53 (18)	C17—C18—H18C	109.5
C1—C2—C14	121.32 (15)	H18A—C18—H18B	109.5
C3—C2—C1	117.64 (16)	H18A—C18—H18C	109.5
C3—C2—C14	120.98 (16)	H18B—C18—H18C	109.5
N1—C26—H26	118.9	O2—C31—C32	115.3 (2)
N1—C26—C27	122.13 (18)	O2—C31—C30	125.46 (19)
C27—C26—H26	118.9	C30—C31—C32	119.3 (2)
C10—C20—H20	107.8	C31—C30—H30	119.5
C10—C20—C22	111.11 (15)	C31—C30—C29	121.0 (2)
C10—C20—C21	111.23 (16)	C29—C30—H30	119.5
C22—C20—H20	107.8	C14—C16—H16A	109.5
C21—C20—H20	107.8	C14—C16—H16B	109.5
C21—C20—C22	110.92 (16)	C14—C16—H16C	109.5
C32—C27—C26	121.01 (16)	H16A—C16—H16B	109.5
C32—C27—C28	119.76 (18)	H16A—C16—H16C	109.5
C28—C27—C26	119.21 (19)	H16B—C16—H16C	109.5
C6—C17—H17	107.3	C23—C24—H24A	109.5
C6—C17—C19	114.19 (16)	C23—C24—H24B	109.5
C6—C17—C18	110.88 (15)	C23—C24—H24C	109.5
C19—C17—H17	107.3	H24A—C24—H24B	109.5
C19—C17—C18	109.68 (16)	H24A—C24—H24C	109.5
C18—C17—H17	107.3	H24B—C24—H24C	109.5
C12—C13—H13	119.0	O4—C41—H41A	109.5
C8—C13—C12	122.04 (17)	O4—C41—H41B	109.5
C8—C13—H13	119.0	O4—C41—H41C	109.5
C10—C9—H9	118.8	H41A—C41—H41B	109.5
C8—C9—C10	122.50 (17)	H41A—C41—H41C	109.5
C8—C9—H9	118.8	H41B—C41—H41C	109.5
O1—C32—C27	122.06 (16)	C38—C37—H37	119.7
O1—C32—C31	118.3 (2)	C36—C37—C38	120.5 (2)
C27—C32—C31	119.67 (19)	C36—C37—H37	119.7
C6—C5—H5	119.0	C27—C28—H28	120.0
C6—C5—C4	122.02 (16)	C29—C28—C27	120.0 (2)
C4—C5—H5	119.0	C29—C28—H28	120.0
C13—C8—C7	120.93 (18)	C30—C29—H29	119.8
C9—C8—C13	118.42 (16)	C28—C29—C30	120.4 (2)
C9—C8—C7	120.65 (18)	C28—C29—H29	119.8
C2—C14—H14	107.4	C8—C7—C4	114.93 (15)
C15—C14—C2	109.56 (16)	C8—C7—H7A	108.5
C15—C14—H14	107.4	C8—C7—H7B	108.5
C16—C14—C2	114.26 (16)	C4—C7—H7A	108.5
C16—C14—H14	107.4	C4—C7—H7B	108.5
C16—C14—C15	110.48 (15)	H7A—C7—H7B	107.5
C12—C23—H23	107.2	C20—C21—H21A	109.5
C12—C23—C25	110.64 (15)	C20—C21—H21B	109.5
C12—C23—C24	113.66 (16)	C20—C21—H21C	109.5
C25—C23—H23	107.2	H21A—C21—H21B	109.5
C24—C23—H23	107.2	H21A—C21—H21C	109.5
C24—C23—C25	110.75 (16)	H21B—C21—H21C	109.5
C5—C4—C7	121.60 (17)	O2—C33—H33A	109.5
C3—C4—C5	118.58 (16)	O2—C33—H33B	109.5
C3—C4—C7	119.80 (18)	O2—C33—H33C	109.5
C39—C38—H38	119.7	H33A—C33—H33B	109.5
C39—C38—C37	120.64 (18)	H33A—C33—H33C	109.5
C37—C38—H38	119.7	H33B—C33—H33C	109.5
			
O3—C40—C35—C34	2.5 (2)	C6—C1—C2—C14	176.57 (15)
O3—C40—C35—C36	−179.12 (16)	C6—C1—C2—C3	−0.7 (2)
O3—C40—C39—O4	−1.1 (2)	C6—C5—C4—C3	−0.3 (3)
O3—C40—C39—C38	179.22 (16)	C6—C5—C4—C7	−178.75 (18)
O1—C32—C31—O2	−1.5 (2)	C35—C40—C39—O4	177.23 (15)
O1—C32—C31—C30	−179.72 (17)	C35—C40—C39—C38	−2.4 (2)
N2—C11—C12—C13	177.89 (15)	C35—C36—C37—C38	−1.8 (3)
N2—C11—C12—C23	−3.2 (2)	C39—C40—C35—C34	−175.79 (15)
N2—C11—C10—C20	−0.9 (2)	C39—C40—C35—C36	2.6 (3)
N2—C11—C10—C9	−178.65 (15)	C39—C38—C37—C36	1.9 (3)
N2—C34—C35—C40	−2.1 (3)	C2—C1—C6—C17	177.89 (15)
N2—C34—C35—C36	179.51 (17)	C2—C1—C6—C5	0.3 (2)
O4—C39—C38—C37	−179.43 (19)	C26—N1—C1—C6	108.15 (19)
N1—C1—C6—C17	−6.6 (2)	C26—N1—C1—C2	−76.2 (2)
N1—C1—C6—C5	175.87 (15)	C26—C27—C32—O1	−0.1 (3)
N1—C1—C2—C14	1.2 (2)	C26—C27—C32—C31	−178.83 (16)
N1—C1—C2—C3	−176.08 (15)	C26—C27—C28—C29	179.62 (18)
N1—C26—C27—C32	0.0 (3)	C20—C10—C9—C8	−175.86 (17)
N1—C26—C27—C28	−178.98 (17)	C27—C32—C31—O2	177.26 (16)
O2—C31—C30—C29	−177.0 (2)	C27—C32—C31—C30	−1.0 (3)
C11—N2—C34—C35	177.84 (15)	C27—C28—C29—C30	−0.6 (3)
C11—C12—C13—C8	−0.1 (3)	C17—C6—C5—C4	−177.33 (17)
C11—C12—C23—C25	−86.5 (2)	C13—C12—C23—C25	92.4 (2)
C11—C12—C23—C24	148.19 (17)	C13—C12—C23—C24	−33.0 (2)
C11—C10—C20—C22	−121.14 (18)	C13—C8—C7—C4	130.3 (2)
C11—C10—C20—C21	114.75 (19)	C9—C10—C20—C22	56.6 (2)
C11—C10—C9—C8	2.0 (3)	C9—C10—C20—C21	−67.5 (2)
C1—N1—C26—C27	179.37 (15)	C9—C8—C7—C4	−49.5 (3)
C1—C6—C17—C19	153.14 (16)	C32—C27—C28—C29	0.6 (3)
C1—C6—C17—C18	−82.3 (2)	C32—C31—C30—C29	1.1 (3)
C1—C6—C5—C4	0.2 (3)	C5—C6—C17—C19	−29.4 (2)
C1—C2—C14—C15	−79.1 (2)	C5—C6—C17—C18	95.1 (2)
C1—C2—C14—C16	156.29 (16)	C5—C4—C3—C2	−0.1 (3)
C1—C2—C3—C4	0.5 (3)	C5—C4—C7—C8	−34.7 (3)
C40—C35—C36—C37	−0.5 (3)	C14—C2—C3—C4	−176.71 (17)
C40—C39—C38—C37	0.2 (3)	C23—C12—C13—C8	−179.00 (17)
C12—C11—C10—C20	173.55 (15)	C3—C2—C14—C15	98.0 (2)
C12—C11—C10—C9	−4.2 (2)	C3—C2—C14—C16	−26.6 (2)
C12—C13—C8—C9	−2.0 (3)	C3—C4—C7—C8	146.9 (2)
C12—C13—C8—C7	178.19 (19)	C31—C30—C29—C28	−0.3 (3)
C10—C11—C12—C13	3.3 (2)	C41—O4—C39—C40	−178.36 (16)
C10—C11—C12—C23	−177.79 (15)	C41—O4—C39—C38	1.3 (3)
C10—C9—C8—C13	1.0 (3)	C28—C27—C32—O1	178.83 (16)
C10—C9—C8—C7	−179.16 (19)	C28—C27—C32—C31	0.1 (3)
C34—N2—C11—C12	109.56 (18)	C7—C4—C3—C2	178.41 (19)
C34—N2—C11—C10	−75.8 (2)	C33—O2—C31—C32	174.40 (18)
C34—C35—C36—C37	177.86 (19)	C33—O2—C31—C30	−7.5 (3)

### Hydrogen-bond geometry (Å, º)

**Table:**

*D*—H···*A*	*D*—H	H···*A*	*D*···*A*	*D*—H···*A*
O3—H3···N2	0.87 (2)	1.79 (2)	2.5912 (17)	153 (2)
O1—H1···N1	0.87 (2)	1.80 (2)	2.5991 (19)	153 (2)
C41—H41*B*···O2^i^	0.98	2.49	3.452 (3)	168

Symmetry code: (i) *x*, *y*, *z*+1.
